# Membrane Composition Modulates Vp54 Binding: A Combined Experimental and Computational Study

**DOI:** 10.3390/pathogens14101000

**Published:** 2025-10-03

**Authors:** Wenhan Guo, Rui Dong, Ayoyinka O. Okedigba, Jason E. Sanchez, Irina V. Agarkova, Elea-Maria Abisamra, Andrew Jelinsky, Wayne Riekhof, Laila Noor, David D. Dunigan, James L. Van Etten, Daniel G. S. Capelluto, Chuan Xiao, Lin Li

**Affiliations:** 1 Department of Pharmaceutical Sciences, University of Texas at El Paso, El Paso, TX 79968, USA; wguo2@utep.edu (W.G.); jesanchez6@miners.utep.edu (J.E.S.); 2 Department of Chemistry and Biochemistry, University of Texas at El Paso, El Paso, TX 79968, USA; rdong@miners.utep.edu (R.D.); lpn5181@psu.edu (L.N.); 3 Department of Biological Sciences, Fralin Life Sciences Institute and Center for Soft Matter and Biological Physics, Virginia Tech, Blacksburg, VA 24061, USAeleamaria@vt.edu (E.-M.A.); andrewj01@vt.edu (A.J.); 4 Nebraska Center for Virology, Department of Plant Pathology, University of Nebraska-Lincoln, Lincoln, NE 68583, USA; irina@unl.edu (I.V.A.); wriekhof2@unl.edu (W.R.); ddunigan2@unl.edu (D.D.D.);; 5 Border Biomedical Research Center, University of Texas at El Paso, El Paso, TX 79968, USA; 6 Department of Computational Science, University of Texas at El Paso, El Paso, TX 79968, USA; 7 Department of Physics, University of Texas at El Paso, El Paso, TX 79968, USA

**Keywords:** membrane–protein interaction, lipid composition, major capsid protein, Vp54, electrostatic features, membrane curvature

## Abstract

The recruitment of peripheral membrane proteins is tightly regulated by membrane lipid composition and local electrostatic microenvironments. Our experimental observations revealed that Vp54, a viral matrix protein, exhibited preferential binding to lipid bilayers enriched in anionic lipids such as phosphatidylglycerol (PG) and phosphatidylserine (PS), compared to neutral phosphatidylcholine/phosphatidylethanolamine liposomes, and this occurred in a curvature-dependent manner. To elucidate the molecular basis of this selective interaction, we performed a series of computational analyses including helical wheel projection, electrostatic potential calculations, electric field lines simulations, and electrostatic force analysis. Our results showed that the membrane-proximal region of Vp54 adopted an amphipathic α-helical structure with a positively charged interface. In membranes containing PG or PS, electrostatic potentials at the interface were significantly more negative, enhancing attraction with Vp54. Field line and force analyses further confirmed that both the presence and spatial clustering of anionic lipids intensify membrane–Vp54 electrostatic interactions. These computational findings align with experimental binding data, jointly demonstrating that membrane lipid composition and organization critically modulate Vp54 recruitment. Together, our findings highlight the importance of electrostatic complementarity and membrane heterogeneity in peripheral protein targeting and provide a framework applicable to broader classes of membrane-binding proteins.

## 1. Introduction

Large double-stranded DNA (dsDNA) viruses often harbor internal lipid membranes that are essential for viral assembly, stability, and host–virus interactions [1,2,3,4,5]. In many systems, the recruitment of peripheral capsid proteins to these membranes is strongly influenced by lipid composition, with anionic lipids frequently enhancing binding affinity through electrostatic attraction [6,7,8,9,10]. Elucidating the physicochemical principles that govern these interactions is critical for understanding viral morphogenesis and may reveal novel targets for antiviral intervention [11,12,13].

The *Paramecium bursaria* chlorella virus-1 (PBCV-1) is a dsDNA giant virus that infects the green alga *Chlorella variabilis* [14,15,16,17,18]. Cryo-electron microscopy (cryo-EM) demonstrated that the 190 nm diameter PBCV-1 virion adopts an icosahedral architecture harboring a multilayer electron-dense core [19]. At 8.5 Å resolution, the virus reveals a unique vertex featuring a spike structure that coats a single lipid bilayer. At 3.5 Å resolution, the capsid is resolved into 30 virus-encoded proteins organized into six distinct classes of capsomers [20]. Each capsomer is organized as a trimer primarily composed of the major capsid protein (MCP; Vp54), with less abundant MCP homologues and additional minor proteins incorporated to decorate the viral capsid [11,21,22,23]. The atomic structures of the MCP has been solved by X-ray crystallography [22,24] and near-atomic-resolution cryo-EM reconstructions [18,25], revealing that the protein adopts two tandem jelly-roll folds [26,27,28].

Beneath the MCP capsid protein layer is a lipid membrane. This lipid bilayer separates the nucleocapsid from the external capsid shell in PBCV-1. The interaction between Vp54 and the lipid bilayer plays a crucial role in maintaining the overall architecture of PBCV-1, as Vp54, a major capsid protein, anchors onto the underlying lipid bilayer to form the continuous icosahedral capsid shell. This protein–lipid interplay not only stabilizes the capsid–membrane junction but also ensures the integrity and infectivity of the virion [8,29,30,31]. Besides Vp54, the minor PBCV-1 capsid proteins P2, P6, P7, P9, P10, P11, P12, P13, and P14 were initially predicted to bear transmembrane regions and, consequently, they could be involved in bridging the external capsid with the internal lipid bilayer [25,32]. For example, the N-terminal region of P9 contains two transmembrane helices that are embedded in the internal lipid bilayer [18]. In addition, one P16 protein, two P17 proteins, and one P18 protein form a transmembrane cluster, whereas P15 makes simultaneous contacts with P9 and P16 and with the lipid bilayer [18].

Despite detailed structural information on PBCV-1, the composition of its internal membrane remain poorly characterized, and the mechanisms underlying Vp54’s lipid selectivity are unknown [1,22]. Lipidomic analysis of *C. variabilis* membranes revealed a predominance of ergosterol and glucosyl inositol phosphoceramide, a plant-like sphingolipid [33]. Infection by PBCV-1 triggers extensive remodeling of the host lipidome, including changes in sterol and sphingolipid biosynthesis that are essential for viral replication and assembly [17,34,35]. PBCV-1 is thought to acquire its internal lipid bilayer from the host, but its precise composition and functional relevance remain to be determined.

Vp54, a major matrix protein of PBCV-1, is a peripheral protein essential for viral assembly and membrane remodeling [22,36,37,38]. Our experimental observations suggested that Vp54 preferentially binds 0.2 mm membranes containing anionic lipids, such as phosphatidylglycerol (PG) and phosphatidylserine (PS), rather than neutral phosphatidylcholine (PC) and phosphatidylethanolamine (PE) membranes. Yet the mechanistic basis of this selective binding remains unclear.

Computational approaches are widely used with experimental methods to study the mechanisms of biomolecule interactions [39,40,41,42,43,44], including the interactions between lipids and proteins [45,46,47,48,49,50]. Here, we investigate how lipid composition influences Vp54 binding by combining experimental membrane-binding assays with atomistic electrostatic modeling and structural analyses. We characterized the amphipathic features of the Vp54 membrane-proximal region, predicted its membrane-binding interface using helical wheel analysis, and evaluated electrostatic potential landscapes, electric field line distributions, and membrane–protein interaction forces for membranes with varying compositions (PC/PE, PG/PC/PE, and PS/PC/PE) using DelPhi Version 7.0 [51] and DelPhiForce C++ V. Beta 1 [52,53]. Our findings reveal that both the charge density and spatial organization of anionic lipids significantly influence Vp54 binding affinity. Electrostatic attraction is strongest when PG or PS is clustered near the binding site, consistent with the experimental observation of enhanced binding in these systems. Together, this work is the first to mechanistically link lipid charge patterning with peripheral protein recruitment, achieved through a synergistic integration of computational modeling and experimental validation. By bridging this key knowledge gap, our study establishes a new framework for understanding how membrane electrostatics govern protein orientation and binding, offering broad implications for lipid–protein interactions in viral assembly and beyond.

## 2. Material and Methods

### 2.1. Chemicals

Dioleoyl phosphatidylcholine (PC), dioleoyl phosphatidylethanolamine (PE), dipamitoyl-sn-glycero-3-phospho-(1’-rac-glycerol) (PG), and dipamitoyl-sn-glycero-3-phospho-L-serine (PS) were obtained from Avanti Research. All other reagents were of analytical grade.

### 2.2. Purification of PBCV-1 Vp54

PBCV-1 virus was propagated according to established procedures and stored in 50 mM Tris-HCl, pH 7.8, at 4 °C [17]. To isolate Vp54 protein, a method developed by Nandhagopal et al. was followed [22]. The virions were heated to 70 °C for 30 min to facilitate capsid protein dissociation and solubilization. Insoluble material was subsequently removed by centrifugation at 1500× *g* for 20 min, yielding a clarified supernatant containing the solubilized Vp54 protein. This clarified supernatant was then subjected to further purification utilizing a HiLoad 26/10 SP Sepharose column (Cytiva, Marlborough, MA, USA) for enhanced resolution and capacity. Prior to sample application, the column was thoroughly equilibrated with filtered and degassed Buffer A, consisting of 50 mM Tris-HCl, 10 mM MgCl_2_, pH 7.8, for a minimum of five column volumes or until the UV absorbance signal stabilized at baseline levels, ensuring complete buffer exchange and optimal column performance. The clarified supernatant containing the target protein was subsequently loaded onto the pre-equilibrated column at a controlled flow rate of 8 mL/min to maintain optimal binding conditions and prevent column overloading. The chromatographic separation was executed using an optimized linear salt gradient elution strategy, progressing from 0 to 100% Buffer B (50 mM Tris-HCl, 1 M NaCl, 10 mM MgCl_2_, pH 7.8) over a predetermined gradient volume to achieve selective elution of Vp54 protein based on its ionic properties. During the purification process, twenty-five discrete fractions were systematically collected across the elution profile and subsequently analyzed by SDS-PAGE to assess protein purity, molecular weight integrity, and overall purification yield. Following chromatographic separation, fractions containing the purified Vp54 protein were pooled, concentrated, and subjected to buffer exchange into sodium phosphate buffer (50 mM Na_3_PO_4_, 10 mM MgCl_2_, pH 7.0) using centrifugal concentrators with a 30 kDa molecular weight cut-off (Cytiva, Marlborough, MA, USA) to prepare the protein in the appropriate buffer system for downstream applications and storage.

### 2.3. Liposome Co-Sedimentation Assay

Lipid films were prepared by mixing a total of 1.6 mg of lipids dissolved in organic solvent at the following compositions [54,55]: PC/PE (79%/21%); PC/PE/PG (71%/19%/10%), and PC/PE/PS (71%/19%/10%). Lipid mixtures were combined in glass vials, briefly vortexed, and sonicated to ensure homogeneity. The solvent was evaporated under a stream of nitrogen gas, and the lipid films were further dried under vacuum in a desiccator for 2 h. The dried lipids were rehydrated with 0.8 mL of pre-warmed buffer containing 50 mM HEPES and 150 mM KCl, pH 7.4, to a final lipid concentration of ~2 mg/mL. Lipid suspensions were incubated in a 67 °C water bath to facilitate hydration, followed by six freeze–thaw cycles (1 min in liquid nitrogen and 3 min at 67 °C) to promote multilamellar vesicle formation. To generate unilamellar vesicles, the lipid suspensions were extruded through polycarbonate membranes with pore sizes of 0.4, 0.2, 0.1, or 0.05 µm using a mini-extruder (Avanti Polar Lipids, AL, USA).

For liposome co-sedimentation assays, 20 µg of protein was incubated with 85 µL of liposomes (2 mg/mL) at room temperature for 1 h. Following incubation, the mixtures were centrifuged at 63,500 rpm for 1 h at 20 °C using a TLA 100 rotor (Beckman Coulter, Inc. CA, USA) to separate membrane-bound (pellet) and membrane-unbound (supernatant) fractions. For each sample, 40 μL of supernatant or pellet was mixed with 10 μL of SDS–PAGE loading dye, briefly spun down, and boiled for 5 min at 95 °C. Forty microliters of each sample were then resolved on a 15% tricine gel. Electrophoresis was performed for 2.5 h at 150 V following an initial 15 min run at 110 V, using a standard SDS-PAGE running buffer system. After electrophoresis, gels were stained with 0.1% Coomassie Brilliant Blue. Band intensities corresponding to protein in the supernatant and pellet fractions were quantified using the Bio-Rad Image Lab software.

### 2.4. Quantification and Statistical Analysis

For all liposome co-sedimentation assays, band intensities from SDS-PAGE gels were quantified using the Bio-Rad Image Lab, and the relative amount of pelleted protein was calculated as a fraction of the total protein. Each experiment was repeated independently at least twice, and for each condition, replicate measurements were averaged. Data are reported as mean ± standard deviation (SD). Statistical comparisons between two groups (e.g., liposomes with and without anionic lipid) were performed using a two-sample Student’s *t*-test with equal variance assumed, implemented in Excel. A *p*-value of less than 0.1 (* *p* < 0.1) or 0.05 (** *p* < 0.05) was considered statistically significant.

### 2.5. Structure Preparation for Computational Study

**Figure 1:**
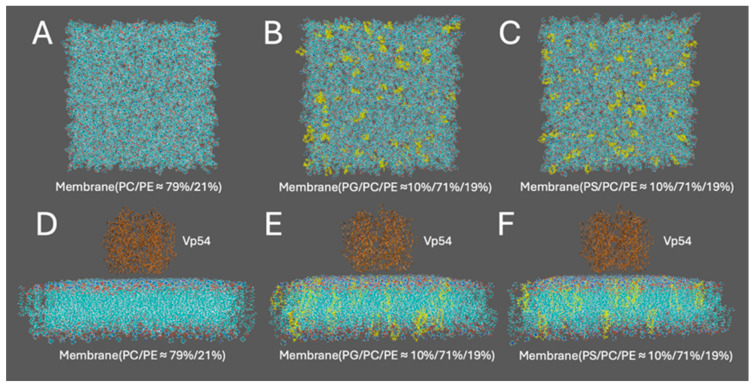
Structure preparation for computational modeling of membrane–Vp54 interactions. (**A**–**C**) Top views of the three membrane models used in the study: (**A**) a zwitterionic membrane composed of PC and PE (79%/21%), (**B**) a membrane with 10% PG evenly distributed within a PC/PE (71%/19%) background, and (**C**) a membrane with 10% PS evenly distributed within a PC/PE (71%/19%) background. (**D**–**F**) Side views of the corresponding systems with Vp54 (brown) placed above the membrane surface. PG and PS are colored yellow in panels (**B**,**C**,**E**,**F**). The membrane is shown in cyan, with anionic lipids highlighted in yellow. Vp54 proteins are shown in orange.

Three membrane systems with distinct lipid compositions were constructed using the CHARMM-GUI Membrane Builder [56]. Each bilayer had a square cross-section of 15 nm × 15 nm. The size chosen was built to avoid periodic boundary condition (PBC) effects in lateral electrostatic calculations. The first system contained a zwitterionic lipid mixture of PC and PE in a molar ratio of approximately 79%/21% (Figure 1A). The other two systems included 10% PG (Figure 1B) or 10% PS (Figure 1C), with the remaining lipids composed of PC (71%) and PE (19%). For both PG and PS systems, configurations with center enrichment of anionic lipids were constructed for further analysis. All membranes featured symmetric leaflets and were hydrated with explicit TIP3P water molecules (≈15–20 Å thickness). The systems were neutralized and ionized with 0.15 M NaCl to replicate physiological ionic strength. The Vp54 protein structure used in this study was extracted from the Protein Data Bank (entry 8H2I) [18]. A single wild-type Major Capsid Protein (MCP; Vp54) was isolated for analysis. The complex structures of the membrane–Vp54 system were constructed using Visual Molecular Dynamics (VMD), with Vp54 initially positioned 4 Å above the membrane surfaces (Figure 1D–F) without any pre-formed contacts or conformational restraints, allowing unbiased evaluation of electrostatic interactions.

### 2.6. Helical Wheel Projection

To evaluate the structural properties of the membrane-proximal region of Vp54, a helical wheel projection was generated using an in-house Python 3.12.11 script. Each residue was assigned an angular position based on the standard α-helical periodicity of 100° per residue and plotted radially to visualize side-chain distribution. The hydrophobic moment (μ) was calculated following the method of Eisenberg et al. [57] by solving the following equation:

(1) $$\mu \left(\delta \right)=\sqrt{{\left(\sum_{i}^{N}H_{i}cos\delta_{i}\right)}^{2}+{\left(\sum_{i}^{N}H_{i}sin\delta_{i}\right)}^{2}}$$

where $H_{i}$ is the hydrophobicity value of residue I; $\delta_{i}$ is the angular position of residue *i* in the helix; and *N* is the total number of residues in the segment. The equation treats each residue as a vector of magnitude $H_{i}$, oriented at an angle $\delta_{i}$ around the helix axis, and computes the magnitude of the resultant vector. This metric reflects the degree of hydrophobic-hydrophilic segregation across the helix and is commonly used to characterize amphipathic α-helices.

### 2.7. Electrostatic Potential Calculations

To study the interaction of the membranes with Vp54, DelPhi Version 7.0 [51] was utilized to calculate electrostatic potential. DelPhi is widely used for calculating the electrostatic potential of biomolecules [58,59,60,61] which solves the Poisson-Boltzmann equation (PBE) as shown in Equation (2) using the finite difference method:

(2) $$\nabla \cdot [\epsilon (r)\nabla \phi (r)]=-4\pi \rho (r)+\epsilon (r)\kappa^{2}(r)sinh(\phi (r)/k_{B}T)$$

where ε(r) is the dielectric permittivity, ϕ(r) is the electrostatic potential, ρ(r) is the permanent charge density, *κ* is the Debye-Hückel parameter, *k_B_* is the Boltzmann constant, and *T* is temperature.

The protein filling percentage of the DelPhi calculation box was set to 70, the probe radius for the molecular structure was 1.4 Å, and the salt concentration was 150 mM, the boundary condition for the PBE was set to the dipolar boundary condition. The calculated electrostatic surface potential was visualized with USCF Chimera [62], with the color scale ranging from −1.0 to 1.0 kT/e.

### 2.8. Electric Field Lines

To gain further insight into the detailed interactions between the membranes and Vp54, electric field lines were calculated using DelPhiForce C++ V. Beta 1 [52,53] and visualized with VMD [63]. The Vp54 was separated by 20 Å from the membranes to get a better visualization. Densities of electric field lines represent the strengths of electrostatic interactions between the membranes and Vp54 in the complex structures. The color scale was set between −1.0 and 1.0 kT/e, corresponding to negative and positive electrostatic potentials, respectively.

### 2.9. Electrostatic Force Calculations

To further investigate the interaction differences between the membranes and Vp54, DelPhiForce C++ V. Beta 1 [52,53] was used to calculate the electrostatic forces for different complex membrane systems [64,65,66]. DelPhiForce is a widely used computational tool for evaluating electrostatic interactions in biomolecular systems. It calculates electrostatic forces by solving the PBE as shown in Equation (2). In this study, the Vp54 was separated from different membranes at varying distances using StructureMan [67]. Specifically, Vp54 was positioned at vertical distances ranging from 10 to 40 Å (2 Å increments) above the membrane center to avoid direct contact while capturing long-range electrostatic effects. Electrostatic forces were then calculated at each separation. To assess the impact of lipid heterogeneity, PG or PS lipids were clustered at the membrane center, and Vp54 was fixed above this enriched region while its vertical position was varied over the same range. Lateral dependence was further examined by holding Vp54 at a constant height (20 Å) and shifting it laterally across the membrane plane (0–50 Å at 5 Å increments), with electrostatic force directions shown as blue arrows. Finally, when PG or PS were clustered at the membrane center, Vp54 was again held at 20 Å above the bilayer and shifted laterally across the membrane plane from 0 Å to 50 Å at 5 Å increments. The directions and strengths of the total electrostatic force were displayed with VMD [63].

## 3. Results and Discussion

### 3.1. PBCV-1 Vp54 Binds Anionic Lipids in a Curvature-Dependent Manner

**Figure 2:**
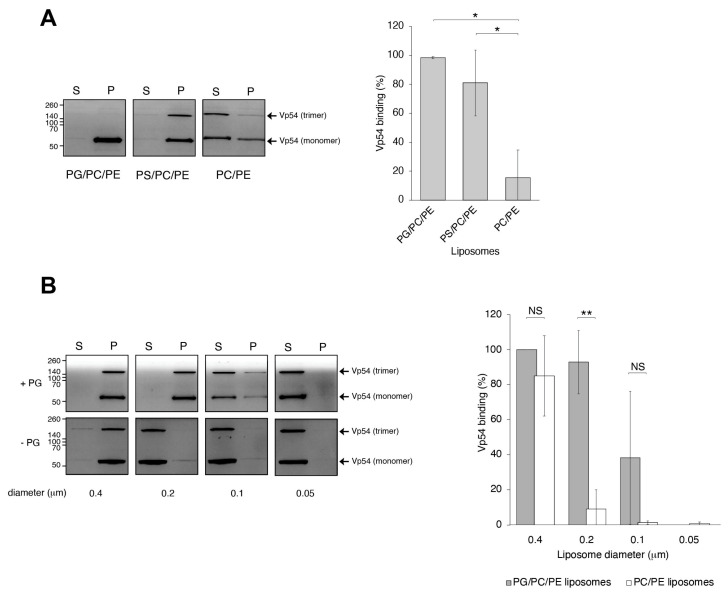
PBCV-1 Vp54 binds anionic lipids in a membrane curvature-dependent manner. (**A**) (**Left**), Representative Coomassie-stained SDS-PAGE of liposome co-sedimentation assays showing Vp54 binding (P, pellet; S, supernatant) to 0.2 μm liposomes composed of PC/PE (zwitterionic), PC/PE/PG (anionic), and PC/PE/PS (anionic). Vp54, free of liposomes, was also fractionated by ultracentrifugation as a control. (**Right**), quantification of Vp54 bound fraction from two independent experiments, plotted as mean ± SD. *, *p* < 0.1. (**B**) (**Left**), as in (**A**) but comparing Vp54 association with PG-containing liposomes extruded through polycarbonate membranes of varying pore sizes (0.4, 0.2, 0.1, and 0.05 μm). (**Right**), Quantification of Vp54 bound fraction from a minimum of three independent experiments, plotted as mean ± SD. **, *p* < 0.05; NS, not significant.

Initial co-sedimentation assays with liposomes of 0.2 μm demonstrated that Vp54 bound significantly to anionic vesicles composed of either PG or PS, when compared with zwitterionic liposomes (Figure 2A,B). Both heat-resistant and heat-labile Vp54 trimers exhibited indistinguishable binding patterns under these conditions. To further evaluate the influence of membrane curvature on Vp54-lipid interactions, liposomes containing PG were extruded through polycarbonate filters of defined pore sizes (0.4, 0.2, 0.1, and 0.05 μm) and incubated with Vp54 in co-sedimentation assays. Vp54 showed nonspecific association with low-curvature, 0.4 μm liposomes, whereas vesicles extruded at 0.2 μm supported markedly enhanced and specific binding relative to both larger (0.4 μm) and smaller (0.1 μm) vesicles (Figure 2A,B). Binding to highly curved, 0.05 μm liposomes was negligible, indicating that excessive curvature impairs Vp54 association (Figure 2B). SDS-PAGE analysis of pellet and supernatant fractions revealed the persistence of Vp54 trimers under denaturing conditions; importantly, both heat-resistant and heat-sensitive trimers displayed the same curvature-dependent binding profile, demonstrating that trimer thermal stability does not modulate membrane curvature preference. Together, these data establish that Vp54 selectively associates with negatively charged membranes in a curvature-dependent manner, with optimal binding to vesicles of approximately 0.2 μm diameter, which is remarkably close to the ~0.19 μm diameter of intact PBCV-1 virions [68], underscoring the physiological relevance of this mechanism.

### 3.2. Helical Projection of the Membrane-Proximal Region

**Figure 3:**
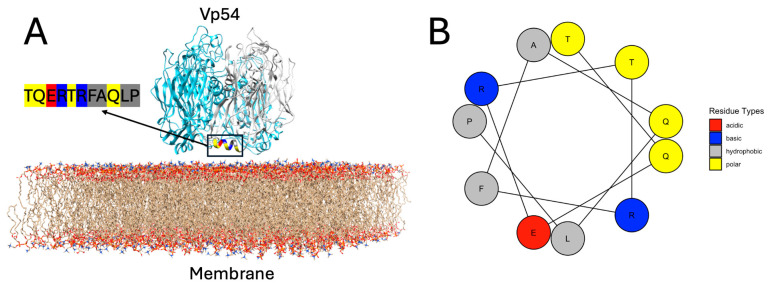
Structural context and helical amphipathicity of the membrane-proximal helix in Vp54. (**A**) Structural model of trimeric Vp54 positioned on a lipid bilayer composed of PC and PE (PC/PE ≈ 79%/21%). One monomer is highlighted in gray, while the other two are shown in blue. The membrane-proximal segment comprising residues 213–223 (TQERTRFAQLP) is highlighted for the gray monomer, with residues color-coded according to types: polar (yellow), basic (blue), acidic (red), and hydrophobic (deep gray). The helix is positioned near parallel to the bilayer surface, with the membrane-facing residues (T213, Q214, R218, F219, L222, and P223) forming a contiguous interaction surface. (**B**) Helical wheel projection of the same segment (residues 213–223), illustrating the amphipathic nature of the helix. Residue types are color-coded as in panel A.

To evaluate the structural properties of the membrane-proximal region of Vp54, a helical wheel projection was constructed for residues 213–223 (TQERTRFAQLP). In Figure 3A, the residues from one representative chain are shown in gray, while the other two chains are colored in cyan, to enhance clarity and focus on the structural features of a single helix. This projection visualizes the spatial distribution of side chains within this α-helical region. The six residues, T213, Q214, R218, F219, L222, and P223, are oriented toward the membrane-facing side of the helix, forming a chemically heterogeneous but spatially contiguous interface. The projection revealed a well-defined amphipathic organization, with most of the polar and charged residues (T213, Q214, and R218) clustering on one side of the helix, and hydrophobic residues (F219, L222, and P223) aligning on the opposite face. Among these, T213 and Q214 are polar, uncharged residues capable of forming hydrogen bonds with lipid headgroups. R218 is positively charged and may contribute electrostatic interactions with negatively charged lipid components. F219, L222, and P223 are hydrophobic and likely engage with the acyl chain region of the bilayer. This spatial arrangement reflects a typical amphipathic α-helix (Figure 3B), in which polar and charged residues are exposed to the aqueous or lipid headgroups, while hydrophobic residues interact with the interior of Vp54. The hydrophobic moment (μ) was calculated to be 6.63, indicating strong segregation of hydrophobic and hydrophilic residues. This level of amphipathicity is characteristic of α-helices that associate with membrane surfaces through selective side-chain partitioning [57].

### 3.3. Electrostatic Potential of Membrane–Vp54 Interfaces

**Figure 4:**
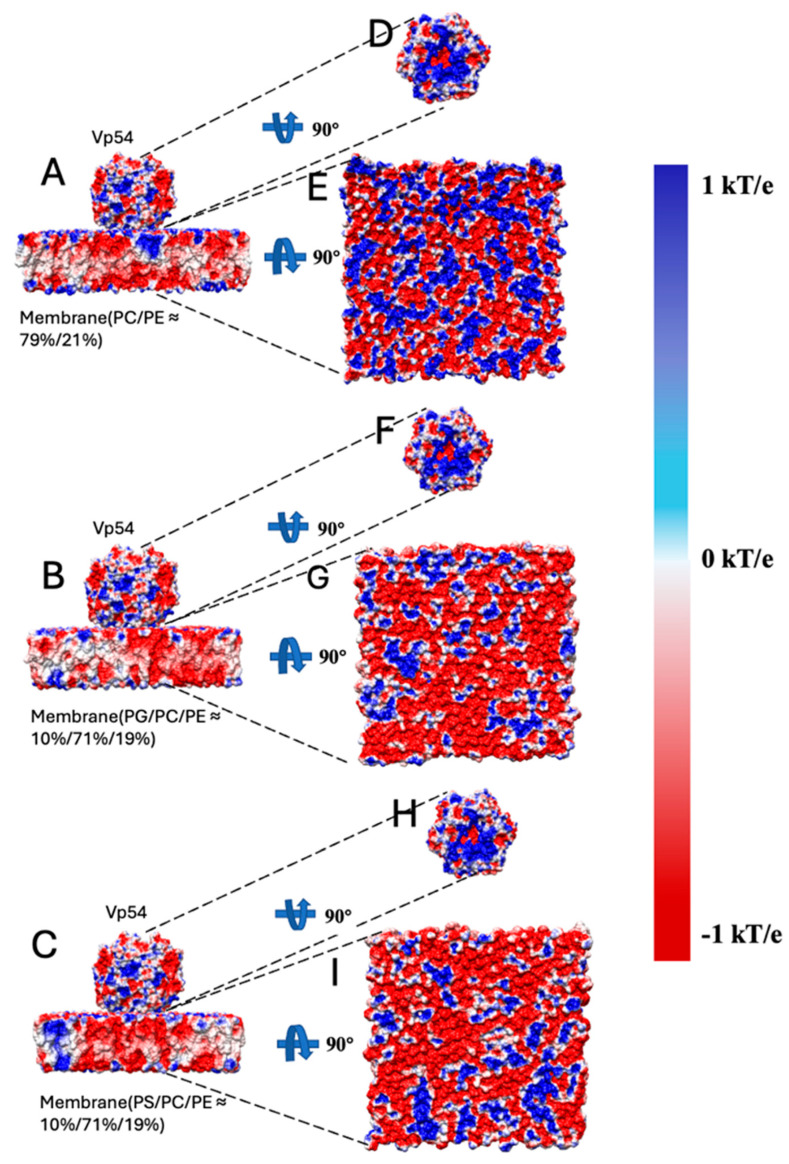
Electrostatic potential distribution of membrane–Vp54 systems with different lipid compositions. Side views of the three modeled systems consisting of Vp54 (top) placed above three membranes: (**A**) PC/PE (79%/21%), (**B**) PG/PC/PE (10%/71%/19%), and (**C**) PS/PC/PE (10%/71%/19%). (**D**–**I**) Electrostatic potential surfaces (mapped in units of kT/e) calculated using DelPhi Version 7.0, shown for the interacting surfaces of Vp54 and the corresponding membranes. Vp54 surfaces (**D**,**F**,**H**) and membrane surfaces (**E**,**G**,**I**) are rotated 90° to reveal the potential distribution at the binding interface. Blue and red indicate regions of positive and negative electrostatic potential, respectively.

The electrostatic potential provides a spatial map of charged regions on biomolecular surfaces, which is essential for understanding how membrane composition influences Vp54 binding. To investigate this, we calculated the electrostatic potential of Vp54 and three membrane models with varying lipid compositions using DelPhi Version 7.0 [51]. The results shown on surfaces, with the structure being rotated at certain angles to show the electrostatic potential on the binding interfaces (Figure 4). In all systems, Vp54 was positioned above the membrane surface in a consistent orientation, and the electrostatic potential was mapped individually for the protein and each membrane. Positively and negatively charged regions are colored in blue and red, respectively. The force between the membrane and the Vp54 is attractive when their interfacial residues possess opposing net charges.

As illustrated in Figure 4A–C, we calculated and showed the side view electrostatic potential of Vp54 and three membrane models with varying lipid compositions. The potential distribution on the Vp54 surface (Figure 4D,F,H) remained consistent across conditions, as expected, since Vp54 was treated identically in all models. Its membrane-facing surface exhibited a prominent positively charged patch, concentrated in the lower helical domain, suggesting a potential site for electrostatic recognition. In contrast, the membrane surfaces displayed composition-dependent electrostatic variations. The PC/PE membrane (Figure 4E) showed a relatively neutral or weakly negative surface, whereas the PG/PC/PE and PS/PC/PE membranes (Figure 4G,I) exhibited stronger negative potentials, particularly in regions enriched with anionic lipids. This enhanced negative surface charge aligns spatially with the positive patch on Vp54, forming a basis for electrostatic complementarity. Electrostatic forces are a major determinant of molecular recognition and binding orientation. The stronger complementarity observed in the PG and PS systems suggests higher binding affinity and stability for Vp54 with membranes containing anionic lipids. These observations are consistent with our electrostatic force calculations and provide a mechanistic explanation for the enhanced membrane association seen in PG- and PS-rich environments. Overall, mapping electrostatic potentials offers critical insight into how lipid composition shapes the interaction landscape.

### 3.4. Electric Field Line Density Reflects Electrostatic Interaction Strength

**Figure 5:**
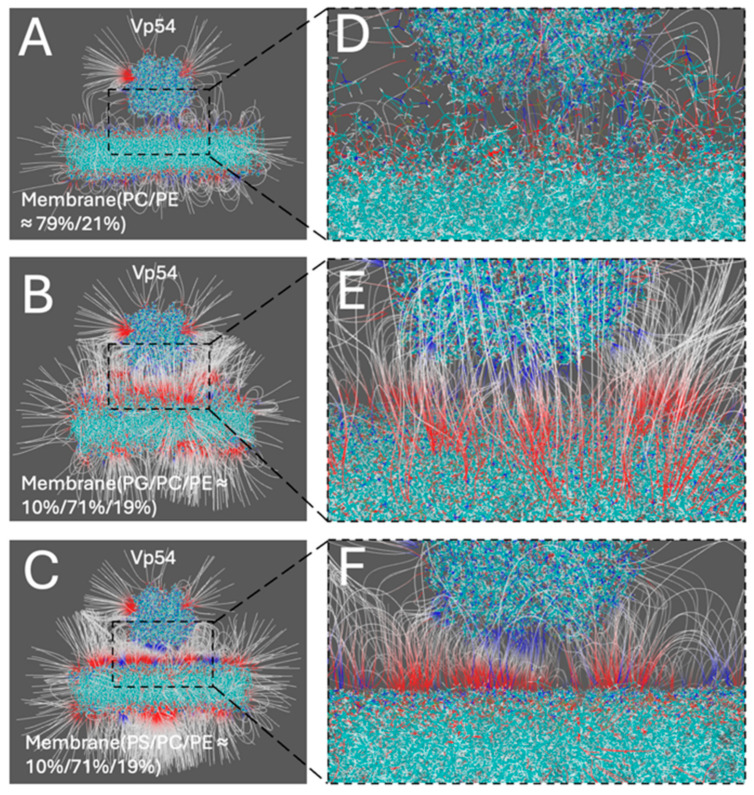
Electric field lines illustrating membrane–Vp54 electrostatic interactions in three different membrane compositions. (**A**–**C**) Side views of the membrane–Vp54 systems showing the electrostatic field lines, composed of: (**A**) PC/PE (79%/21%), (**B**) PG/PC/PE (10%/71%/19%), and (**C**) PS/PC/PE (10%/71%/19%). Vp54 (top) is separated from membranes by 20 Å to get a better visualization. (**D**–**F**) Zoomed-in views of the membrane surface region corresponding to (**A**–**C**), showing the spatial distribution and density of field lines near the membrane–Vp54 interface. Electric field lines color scale was set between −1.0 and 1.0 kT/e, corresponding to negative and positive electrostatic potentials, respectively.

To evaluate the relative strength and spatial orientation of electrostatic interactions between Vp54 and membranes of different compositions, electric field lines were calculated for each system using DelPhiForce C++ V. Beta 1. Figure 5A–C shows the side views of three membrane–Vp54 complexes, consisting of (A) PC/PE (79%/21%), (B) PG/PC/PE (10%/71%/19%), and (C) PS/PC/PE (10%/71%/19%). In each case, Vp54 was placed 20 Å above the membrane surface in a non-contacting orientation to get a better visualization. Figure 5D–F provides zoomed-in views of the membrane surface region corresponding to each system. The density of electric field lines provides an intuitive visual indicator of the strength of electrostatic interactions: higher line density corresponds to stronger electrostatic forces acting between the membrane and Vp54. In the PC/PE system (Figure 5A,D), the field lines are sparse and disorganized, indicating weak and diffuse electrostatic interactions. By contrast, the PG- and PS-containing systems (Figure 5B,C,E,F) exhibit markedly denser and more coherent field lines, particularly in the region directly beneath Vp54, suggesting stronger and more focused electrostatic attraction. These observations align with electrostatic potential surface analysis (Figure 4) and provide spatial evidence that anionic lipid enrichment enhances the electrostatic pull experienced by Vp54. Together, these field line visualizations reinforce the conclusion that electrostatic interaction strength is lipid-composition-dependent, and that PG and PS facilitate stronger and more directed attraction toward Vp54 than zwitterionic membranes.

### 3.5. Lipid Composition and Spatial Dependence of Electrostatic Forces

**Figure 6:**
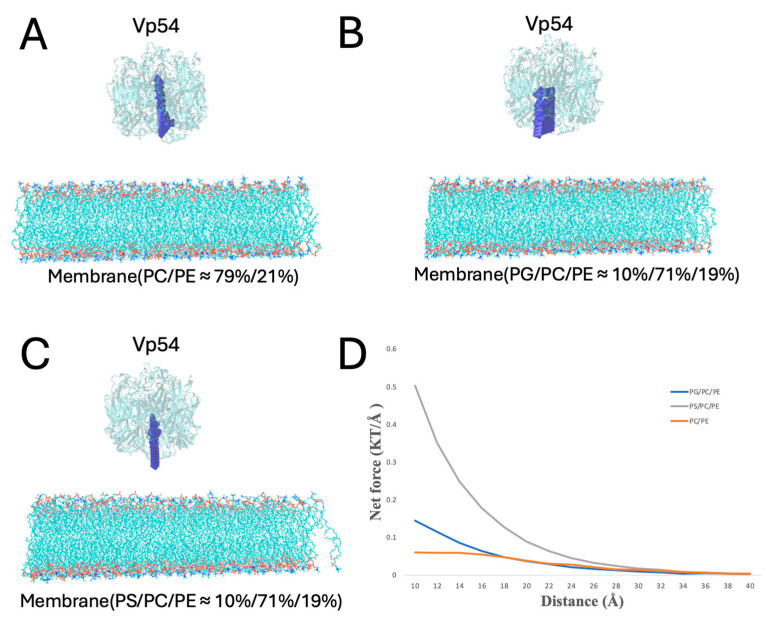
Electrostatic force analysis between Vp54 and membranes of different compositions. (**A**–**C**) Side views of the membrane–Vp54 systems with Vp54 positioned 20 Å above the membrane surface for electrostatic interaction calculations. The three membrane types include: (**A**) PC/PE (≈79%/21%), (**B**) PG/PC/PE (10%/71%/19%), and (**C**) PS/PC/PE (10%/71%/19%). (**D**) Magnitude of total electrostatic force (kT/Å) between the membrane and Vp54 at distances from 10 to 40 Å with a step size of 2 Å. The blue arrows demonstrate the directions of electrostatic forces.

**Figure 7:**
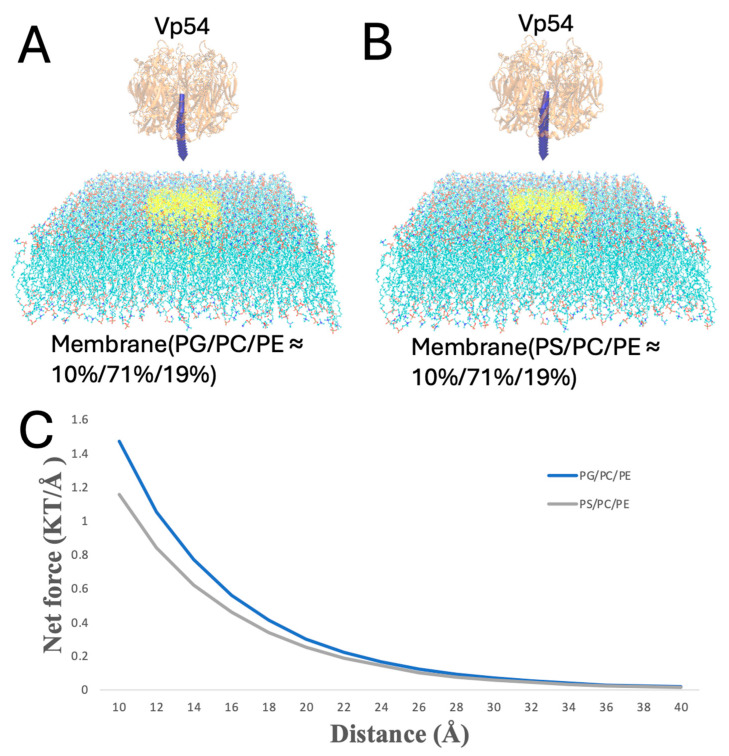
Enhanced electrostatic attraction in membrane–Vp54 systems with centrally positioned anionic lipids. (**A**,**B**) Side-view representations of the membrane–Vp54 systems containing: (**A**) PG/PC/PE (10%/71%/19%) and (**B**) PS/PC/PE (10%/71%/19%), where PG or PS lipids are selectively positioned at the center of the membrane (highlighted in yellow). (**C**) Magnitude of electrostatic force (kT/Å) between the membrane and Vp54 at distances from 10 to 40 Å with a step size of 2 Å. The membrane is shown in cyan, with anionic lipids highlighted in yellow and concentrated at the membrane center. Vp54 proteins are shown in orange.

**Figure 8:**
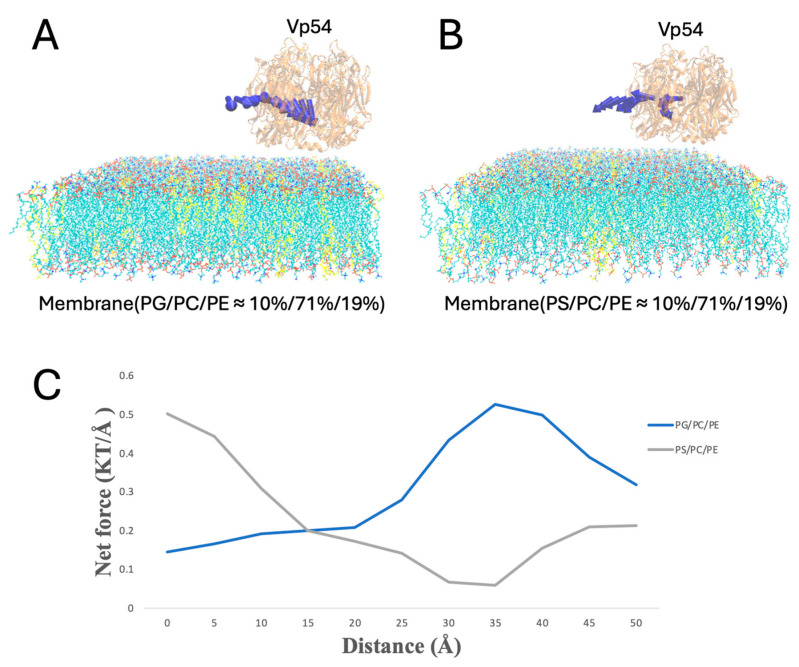
Electrostatic force variation as Vp54 moves from membrane center to edge in systems with evenly distributed anionic lipids. (**A**,**B**) Side views of membrane–Vp54 systems containing evenly distributed anionic lipids (highlighted in yellow): (**A**) PG/PC/PE (10%/71%/19%) and (**B**) PS/PC/PE (10%/71%/19%). Vp54 is positioned at a fixed vertical distance above the membrane surface and systematically moved laterally from the center toward the membrane. (**C**) Magnitude of electrostatic force (kT/Å) between the membrane and Vp54 at distances from 0 to 50 Å with a step size of 5 Å. The membrane is shown in cyan, with anionic lipids highlighted in yellow and distributed evenly. Vp54 proteins are shown in orange.

**Figure 9:**
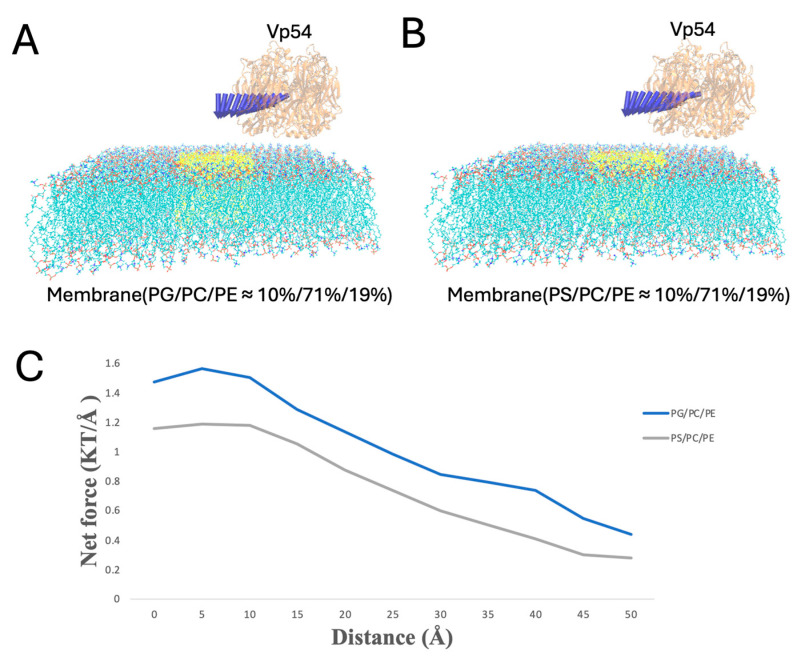
Electrostatic force variation as Vp54 moves laterally from center to edge above membranes with centrally clustered anionic lipids. (**A**,**B**) Side views of membrane–Vp54 systems in which anionic lipids (highlighted in yellow) are concentrated at the membrane center: (**A**) PG/PC/PE (10%/71%/19%) and (**B**) PS/PC/PE (10%/71%/19%). Vp54 is positioned at a fixed vertical distance above the membrane surface and systematically moved laterally from the center toward the membrane edge. (**C**) Magnitude of electrostatic force (kT/Å) between the membrane and Vp54 at distances from 0 to 50 Å with a step size of 5 Å. The membrane is shown in cyan, with anionic lipids highlighted in yellow and concentrated at the membrane center. Vp54 proteins are shown in orange.

To systematically evaluate how lipid composition and spatial positioning affect the electrostatic interactions between Vp54 and lipid membranes, we performed DelPhiForce [52,53]-based electrostatic force calculations across four structural configurations (Figure 6, Figure 7, Figure 8 and Figure 9). The non-electrostatic forces, such as van der Waals interactions, also contribute to protein–membrane interactions. However, the van der Waals force decreases much more dramatically compared to the electrostatic force, due to the Lennard-Jones equation. In this study, we investigated the long-range force when the Vp54 was separated from the lipids at 10–50 Å. At this range, the electrostatic force was dominant while the van der Waals and other forces could be ignored. In each system, PG or PS lipids (10%) were incorporated into a PC/PE (71%/19%) background either evenly or concentrated at the membrane center. Vp54 was positioned above the membrane and translated either vertically (changing distance) or laterally (from center to edge), depending on the setup. The directions of the electrostatic forces between the membrane and the Vp54 are shown in the form of blue arrows. Note that the blue arrows only demonstrate the directions of electrostatic forces because the arrows are normalized to the same size.

In Figure 6, Vp54 was placed at varying vertical distances (from 10 to 40 Å, at 2 Å increments) above the membrane center to avoid direct contact while capturing long-range electrostatic effects (Figure 6A–C). The electrostatic forces between the membrane and the Vp54 were then calculated at each separation by DelPhiForce C++ V. Beta 1 [52,53]. According to the direction of the blue arrows, the PG- and PS-containing membranes exhibit attraction across all distances, while the PC/PE membrane shows repulsive forces. Figure 6D shows the magnitude of the total electrostatic force (in kT/Å) as a function of distance. The electrostatic forces of the membrane–Vp54 decreased as the distance between the membrane–Vp54 increased, which is expected from Coulomb’s law. The PS-containing system exhibited the strongest electrostatic attraction across all distances, followed by the PG-containing membrane, while the PC/PE membrane produced the weakest interaction. This hierarchy of interaction strength directly corresponds to the anionic lipid content, with PS exerting a more pronounced effect than PG at equivalent molar percentages. The difference in calculated forces between PS- and PG-containing membranes likely reflects variations in lipid headgroup structure and charge positions. While both lipids are anionic and interact with the positively charged patch on Vp54, subtle differences in headgroup orientation, spacing, and local packing can modulate the magnitude and direction of electrostatic forces, resulting in the observed divergence. Notably, the force profiles for PG and PS decay more gradually compared to the neutral membrane, suggesting more stable and sustained electrostatic engagement. These results confirm that lipid composition directly modulates the strength and persistence of membrane–Vp54 interactions. These results reinforce our previous observations from electrostatic potential surfaces (Figure 4) and electric field line distributions (Figure 5), confirming that anionic lipid enrichment enhances long-range electrostatic attraction between Vp54 and the membrane. The stronger force profiles of PG and PS systems support their proposed roles in facilitating membrane association of Vp54 and suggest a lipid composition-dependent mechanism for its targeting.

In Figure 7, PG or PS lipids are clustered at the membrane center, and Vp54 is fixed directly above this enriched region while its vertical position varies (from 10 to 40 Å, at 2 Å increments). Force magnitudes were higher than in the evenly distributed case, and PG produced a stronger and more sustained force profile compared to PS. This suggests that spatial clustering of anionic lipids further amplifies the interaction strength, likely by reinforcing localized electrostatic fields beneath the protein.

To evaluate lateral dependence, Vp54 was held at a fixed height above the membrane (20 Å) and shifted along the membrane (from 0 to 50 Å, at 5 Å increments) (Figure 8A,B); the blue arrows indicate the direction of electrostatic forces between Vp54 and the membrane at different positions, which showed no consistent pattern. In this configuration with evenly distributed anionic lipids, Figure 8C shows that the total electrostatic force showed no consistent pattern across lateral positions for either PG- or PS-containing membranes. Although PG generated slightly higher forces overall, the force magnitudes fluctuated irregularly along the membrane surface, indicating that no clear lateral preference was observed in the absence of lipid clustering. These results suggest that even distribution of anionic lipids fails to generate strong directional cues, and spatial localization (Figure 9) may be essential for focusing electrostatic attraction.

Finally, when PG or PS clustered at the membrane center, Vp54 was held at a fixed height above the membrane (20 Å) and shifted along the membrane (from 0 to 50 Å, at 5 Å increments) (Figure 9). The force peaked directly over the central lipid and decreased toward the edge. PG produced higher force magnitudes throughout. These findings suggest that Vp54 is more strongly guided toward membrane regions enriched in PG/PS, consistent with earlier electrostatic potential and field line analyses.

Together, these force analyses reveal that membrane–Vp54 electrostatic interactions are strongly influenced by both lipid composition and spatial organization. The presence of anionic lipids-PG/PS-significantly enhances electrostatic attraction, and this effect is further amplified when these lipids are spatially clustered within the membrane. Additionally, the strength and directionality of the forces are highly sensitive to both vertical distance and lateral positioning of Vp54 relative to the membrane surface. These findings underscore the functional importance of lipid microdomains in guiding peripheral protein recruitment and orientation, a principle that may extend to a wide range of membrane-associated proteins beyond Vp54.

## 4. Conclusions

In this study, we investigated how membrane composition influences the electrostatic recognition and binding behavior of Vp54, a peripheral membrane protein. Through a combination of experimental and computational modeling validation, we showed that both lipid identity and spatial distribution critically shape membrane–protein interactions.

Experimental data of membrane binding assays revealed that Vp54 preferentially associates with PG- and PS-containing vesicles in a curvature-dependent manner. PG/PC/PE and PS/PC/PE mixtures were used as simplified model membranes. Although *C. variabilis* lipids contain ergosterol and glucosyl inositol phosphoceramide [69,70], these model systems provide well-characterized, reproducible environments to probe fundamental lipid–protein interactions. Future studies will include other lipids in modeled membranes to more closely mimic the host-derived viral membrane. Our data also indicate that Vp54 binding is reduced on highly curved membranes (e.g., 0.05 µm liposomes). This likely reflects curvature-induced changes in lipid packing and electrostatic organization, which decrease the effective surface density of accessible anionic headgroups and reduce the ability of Vp54 to engage the lipid bilayer.

The computational findings validated the experimental data. Note that our analysis is based on rigid structures of Vp54 and lipid membranes. Membrane dynamics and protein flexibility could modulate binding orientation and interaction strength, while the presence of diverse host lipids may affect local charge distribution and lipid packing. Consequently, these factors could significantly influence Vp54 recruitment and stability, and future studies incorporating full membrane complexity and protein flexibility would provide a more comprehensive understanding. Structurally, the membrane-proximal region of Vp54 adopts a highly amphipathic α-helical conformation (residues 213–223), with positively charged and hydrophobic residues forming a well-defined interface favorable for membrane engagement. This architecture provides a physicochemical basis for selective interactions with negatively charged lipids. Electrostatic potential analysis revealed that membranes containing anionic lipids (PG or PS) present more negative surface potentials compared to zwitterionic PC/PE membranes. While Vp54’s interface remains positively charged across systems, the enhanced charge complementarity in PG- and PS-containing membranes supports stronger binding. Electric field line analysis further demonstrated that field density near the interface correlates with interaction strength, high in PG and PS, and minimal in PC/PE control. Quantitative force calculations confirmed that PS/PG-rich systems exhibit a strong electrostatic attraction, particularly when the anionic lipids are spatially clustered, underscoring the importance of membrane heterogeneity in modulating peripheral protein affinity. We acknowledge that non-electrostatic forces, such as van der Waals interactions, also contribute to protein–membrane binding; however, these forces decay much more rapidly than electrostatics according to the Lennard-Jones potential. As our analysis focuses on the 10–50 Å separation range, electrostatic interactions are dominant while van der Waals contributions can be reasonably neglected.

Together, this combined experimental and computational analysis demonstrates that Vp54 membrane binding is governed not only by lipid charge but also by lateral lipid organization. This work highlights the broader principle that electrostatic microenvironments within biological membranes can modulate protein recruitment, orientation, and function. The concept has implications for viral protein targeting, host–pathogen interactions, and the design of membrane-active therapeutics. These findings have broader implications for peripheral membrane protein recruitment and organization. The pronounced amphipathicity and positively charged patch on Vp54 illustrate how electrostatic complementarity with anionic lipid surfaces can guide both binding orientation and membrane association. Similar mechanisms may apply to other peripheral membrane proteins, where specific charge distributions and amphipathic regions facilitate selective recruitment, stabilize membrane interactions, and potentially influence the spatial arrangement of multiple proteins on the membrane surface. This highlights a general principle by which membrane composition and protein surface properties together shape the organization and function of peripheral membrane assemblies.

## Data Availability

The original contributions presented in this study are included in the article. Further inquiries can be directed to the corresponding authors.
